# Perceived differences in coastal tourism image under tourist experience-IPA analysis based on UGC data of 12 coastal cities

**DOI:** 10.1371/journal.pone.0299431

**Published:** 2024-08-22

**Authors:** Meng Zhang, Qianbin Di, Ying Liu

**Affiliations:** 1 Institute of Marine Sustainable Development, Liaoning Normal University, Dalian, China; 2 School of Geography, Liaoning Normal University, Dalian, China; Rikkyo University, JAPAN

## Abstract

The destination image perceived by tourists is crucial to coastal tourism market positioning and marketing. This paper utilizes tourists’ Internet-generated content from 2017–2021, adopts the jieba text analysis method to identify the cognitive, emotional, and overall image of coastal tourism, divides the constituent elements of the destination image into four main classes and 20 subclasses through the text clustering method, and explores the tourists’ perception of the image of coastal tourism with the help of the IPA model. The study found that: 1) The commonality of the cognitive image of “ocean” in 12 coastal cities is outstanding, but the internal characteristics are obvious, tourists pay more attention to coastal tourism in Bohai Rim and southern coastal areas, and Shanghai, Ningbo and Hangzhou show strong correlation; 2) Tourists’ emotional image of coastal tourism destinations is dominated by positive attitudes, with a high overlap of adjectives representing positive emotions, but with heat differences in different cities; 3) The overall image of coastal tourism can be divided into three circles, including “traditional core-characteristic structure-peripheral perception”, and there are obvious differences in the characteristics of the social semantic network of each city; 4) Tourists are more satisfied with the components of coastal tourism image, but pay more attention to the construction of optimized coastal tourism environment. Based on this, in the process of coastal tourism development, it is necessary to focus on creating distinctive and diversified tourism values, focusing on tourists’ experience needs, improving the construction of quality tourism facilities and services, and promoting the high-quality development of coastal tourism.

## 1. Introduction

Coastal tourism is mainly based on coastal zones, islands, and marine landscapes which offer resources for marine sightseeing tours, recreation, vacation and yacht cruising, and other activities [1]. With the unique location advantages, a high degree of openness, and active tourism investment attraction, coastal tourism has become a real marine pillar industry [2]. The Outline of the Twelfth Five-Year Plan for National Economic and Social Development of the People’s Republic of China clearly points out that one of the main directions for promoting the development of the marine economy is to “accelerate the development of coastal tourism [3]”. Moreover, the report of the Twentieth National Congress of the Party of the People’s Republic of China in 2022 emphasizes the concept of “building a strong marine country [4]” and the coastal tourism industry is to play its role as “ballast”, injecting new impetus for regional economic development. Tourism experience in the new era pays more attention to the tourists’ needs and feelings, in fact, the 14th Five-Year “Tourism Development Plan” emphasizes the promotion of high-quality development of the tourism industry should focus on the demand side of the management, in order to meet the people’s growing demand for a better life. People-oriented, intelligent, and modernized is the inevitable trend for coastal cities to build a high-quality tourism market system, which requires multi-dimensional efforts in tourism image, product, and service construction.

Good destination image is the soul and core of creating a market IP to introduce, stay, and visit, and can enhance the attachment and loyalty of tourists [5], create a word-of-mouth effect, and promote the development of tourism value [6]. Coastal cities in homogeneous tourism resources should be based on tourism resource endowment, closely linked to the consumer market frontier, systematic research, scientific positioning of tourism destination image, characterization, and differentiation to expand their competitive advantage [7]. Research on the differences in tourism image of coastal cities helps to comprehensively recognize the tourism characteristics and advantages of coastal cities and provides scientific guidance and support for the development of coastal tourism. Based on this, this paper explores the characteristics and differences of perceived image and satisfaction of coastal tourism from the perspective of tourists’ experience, with the aim of providing references for coastal cities to reach their target market, grasp the deep needs of tourists, and explore innovative development modes and strategies, so as to enhance the competitiveness and sustainable development of coastal tourism.

## 2. Research progress and theoretical basis

### 2.1 Status of research

With the continuous development of coastal tourism, scholars have carried out useful discussions around coastal tourism, focusing on the development and protection of coastal tourism resources [8], coastal tourism development strategy [9], coastal tourism development influencing factors [10,11], the efficiency of coastal tourism development [12], the loyalty of coastal tourism brand customers [13], the construction of the attractiveness of the coastal city [14], the development of coastal tourism management [15], and sustainable development [16]. Related previous research mostly explores the development strategy and economic value of coastal tourism from the perspective of the destination’s natural landscape conditions [17,18] and management [19]. Only a few scholars have focused on the coastal tourism image from the tourists’ perspective; in addition, they have only considered a single city [20]. However, tourism activities rely on the presence of tourists. The key is to enhance the destination’s attractiveness to tourists, as indicated by research on tourist behavior and perception. Moreover, tourism activities in a relatively static geographical environment are always chasing the most fashionable hotspots, which undergo many changes and need to be constantly updated depending on the demand.

The notion of “destination image” was first proposed in the 20th century, but the academic community has not yet reached a unified definition. Scholars have focused on the internal structure of destination image and proposed several models, including the triad model, cognitive-emotional-whole latitude model, feature model, long-tail model, and core edge-model [21]. In addition, scholars have discussed the factors affecting the destination image [22] and its structure [23], and they have refined tourism activities carried out at the time nodes [24], the city [25], scenic spots [26], the tourism industry [27], and tourist groups. In the research methodology, text mining [28], theme modeling [29], sentiment analysis [30], media recognition [31–33], and other content analysis methods are mainly employed, and the research is completed with the help of technical tools, such as natural language processing technology, LDA modeling, deep learning, and Python [34].

To summarize, the theory of destination image structure has laid a solid theoretical foundation for the study of destination image, and scholars have conducted useful research on coastal tourism and destination image, but there are still the following shortcomings: ①Fewer scholars have started from the perspective of constructing destination image for the development of coastal tourism, and have neglected the role of perception of the main body of tourists, and the same field of research needs to be further updated. ②Fewer scholars have grasped and analyzed the differences between destinations with homogeneous coastal tourism resources in selecting case sites. ③The current analysis of the image of coastal tourism stops at exploring the projected image of the destination, but not at the depth of the perceptual effect on tourists. Based on this, this paper starts from the perspective of tourists’ perception, analyzes the image of coastal tourism from the perspective of tourists and the internal differences of each city, and deeply studies the satisfaction of tourists.

### 2.2 Theoretical foundations

**Fig 1:**
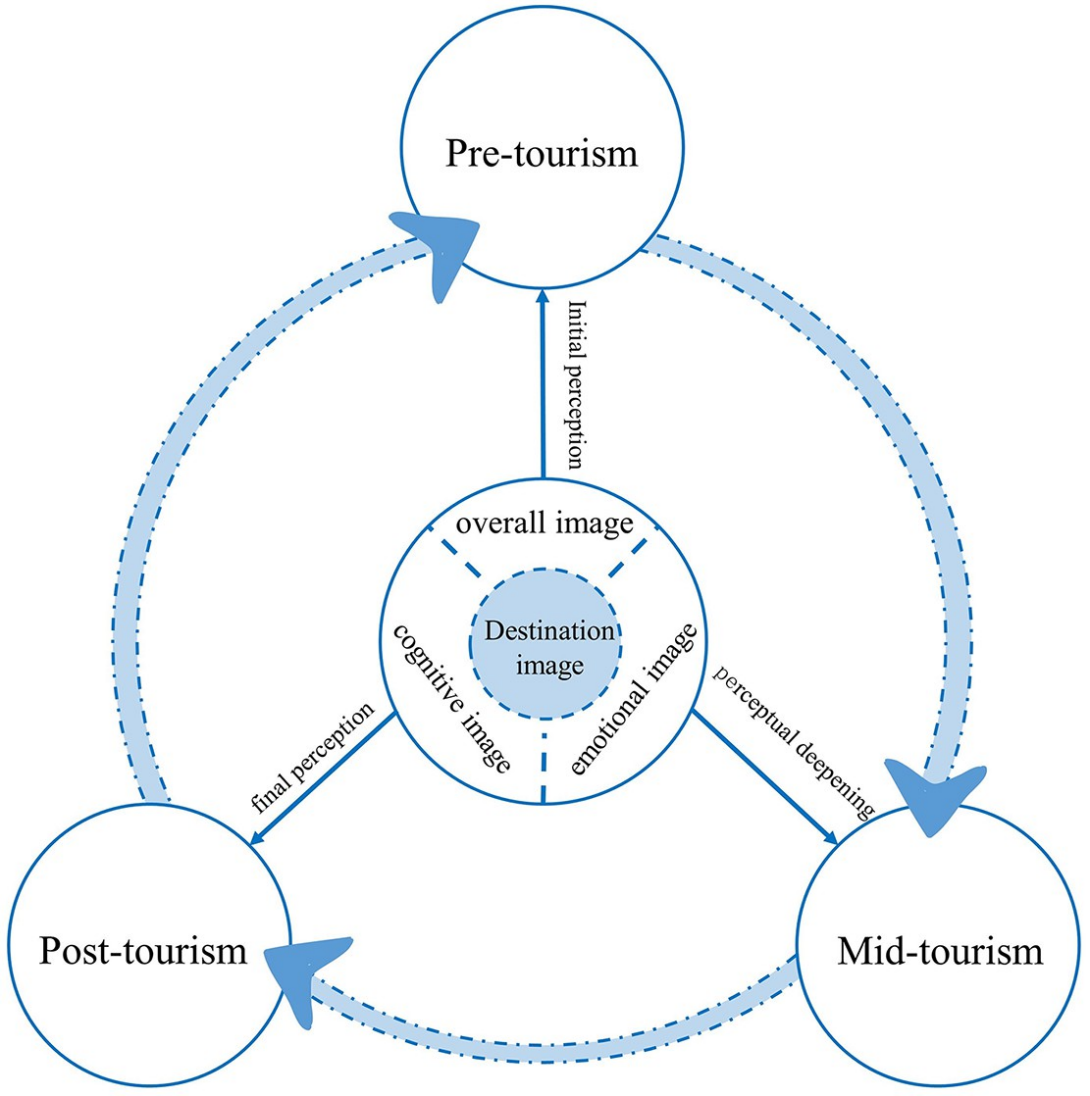
Target terrain image perception model.

The “cognitive-emotional” theory in psychology points out that cognition is the basis for the emergence of emotion, with the latter being the derivation of cognitive concepts [35]. On this basis, Baloglu et al. constructed the “cognitive-emotional-integral” dimensional model [36]. In the Internet era, through online platforms, destinations project a clear destination image to tourists; their perceptions become a mapping of the destination image, which are independent of each other and influence each other, and subconsciously influence tourists’ pre-travel, in-travel and post-travel stages. In the pre-tourism stage, potential tourists pay attention to the destination through the Internet collection and produce preliminary perception; in the mid-tourism stage, the perception will be deepened or changed through the travel experience; in the post-tourism stage, the final perception formed by the tourists not only affects their attachment but also becomes a reference for their potential perception (Fig 1). This tourism cognitive model is widely applied in the cognition of coastal tourism image, particularly in the context of increased interaction between tourists and the destination in the era of big data. Researchers often use this theory in studies focusing on the perception of coastal tourism image in a single coastal tourist city [37]. By exploring the formation and influencing factors of the cognitive image of coastal tourism, researchers analyze tourists’ emotional experience, satisfaction, and behavioral motivations using methods such as the destination emotional dimension scale [38]. The aim is to provide suggestions for the rational development and enhancement of the image of coastal tourism destinations.

## 3. Materials and methods

### 3.1 Study area

There are 54 cities at the prefecture level and above (as of the end of 2021) distributed along the coastline of mainland China with a total length of about 18,000 kilometers, which possess rich and diverse coastal tourism resources with distinctive characteristics. However, only some of them can develop tourism as a representative and leading industry [39]. In order to enhance the research capabilities and consider the accessibility of data, this study selects 12 representative and typical coastal cities such as Dalian, Tianjin, Qingdao, Hangzhou, Ningbo, Shanghai, Fuzhou, Guangzhou, Shenzhen, Xiamen, Zhuhai, and Haikou as the study regions based on the statistical results of inbound tourism foreign exchange earnings and inbound tourism of major cities in the China Tourism Statistical Bulletin published by the National Tourism Administration in 2022. Geographically, these 12 cities have a wide distribution, which covers the main coastal tourism belt in China and can represent the main types of coastal tourism products in the country. In terms of city tourism status, these 12 cities are among the central cities of regional tourism in terms of city grade, tourism income, and tourism reception. They are more representative and comparable regarding tourism resources, transportation, and business management [40]. At present, competition in regional tourism is becoming increasingly fierce and coastal towns are facing major challenges in developing tourism resources and competing for the source market [41]. Using these cities as samples to study the perceived differences in the image of China’s coastal tourism network, this research can reflect the basic situation of the image of major coastal tourism cities.

### 3.2 Research methodology

#### 3.2.1 Evaluation of coastal tourism network attention

In the Internet era, tourists will screen their destination through the network before traveling. Among the many search engines, the Baidu index can quickly and accurately obtain the network attention and trend graphs of coastal cities in different periods, which can directly measure the tourists’ preference for coastal tourism destinations and the destination’s popularity. Referring to the calculation method of Tian Fengjun et al. on the tourism network attention of each city in Jiangxi Province [42], we obtained the tourism network attention of 12 coastal cities. Firstly, the keywords “city name + tourism” (Example: “Dalian tourism”, “Qingdao tourism”) were searched in “Baidu index” to collect data on the daily network attention of the 12 coastal cities from January 1, 2017, to December 31, 2021. Then, with the help of the “average value” function of Baidu Index we obtained the annual daily average value of each coastal city, and through the calculation of the formula (1) we obtained the annual network attention of each coastal city, that is, the city’s tourism popularity on the Internet. The specific formula is:

$$P_{a}=\frac{\left(A_{2017}+A_{2018}+A_{2019}+A_{2020}+A_{2021}\right)}{5}\times 365$$ (1)

Where, *P*_*a*_ denotes the tourism visibility of a coastal city on the Internet; *A*_2017_ denotes the average daily online attention of the city in 2017.

#### 3.2.2 Text analysis method

Text analysis is the most commonly used method in natural language processing and research, including word separation, keyword extraction, sentiment evaluation, and text clustering. The use of text analytics involves roughly the following process: Firstly, a big data information collection system is utilized to collect the content of the comments posted by tourists on the tourism portal and establish an information database. Second, the collected text is subdivided and summarized.As the basis of text analysis, positive or negative results directly affect the confidence and accuracy of text processing [43], so this paper chooses the jieba Chinese participle tool component under Python software to accomplish this task [44]. The collected samples are preprocessed to eliminate repetitive and meaningless words, update the user-defined word list, merge synonyms, and extract high-frequency feature words from the UGC corpus of each city to analyze the cognitive image and emotional image of the image of coastal tourism destinations. Again, the social semantic network graph of tourism image of 12 coastal cities is constructed to recognize their overall image. Finally, with the intelligent annotation function provided by Baidu’s open-source platform EasyData, artificial-assisted machine learning is used to cluster the collected samples [45] and classify the perceived dimensions of the image of coastal tourism destinations.

#### 3.2.3 IPA analysis

**Fig 2:**
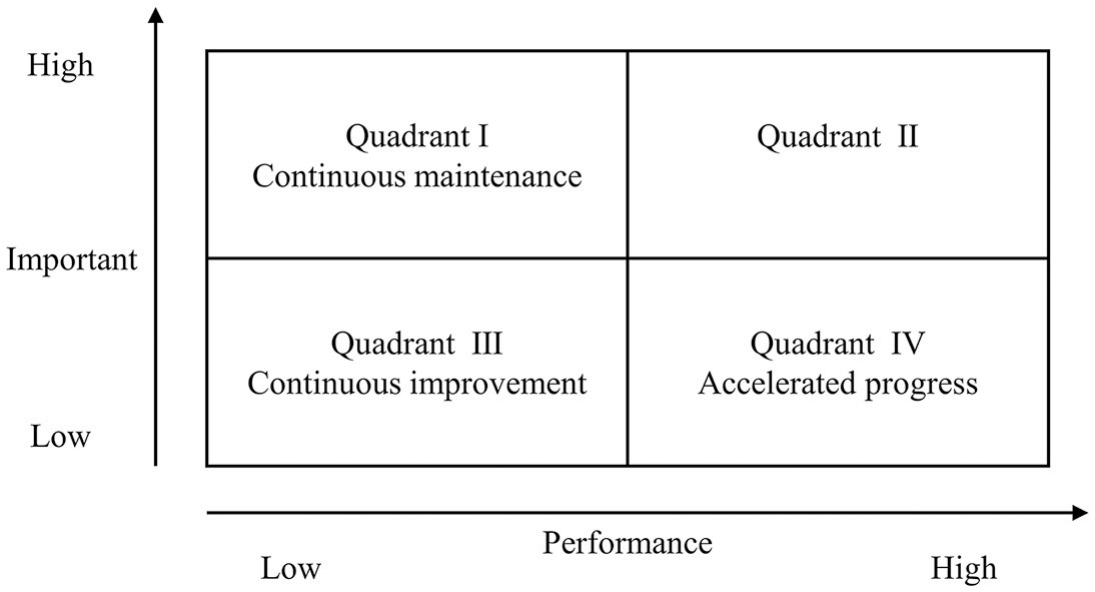
IPA model schematic diagram.

IPA Analysis (Importance-Performance Analysis, IPA), a multi-factor contribution model, was introduced into tourism research at the end of the 20th century [46], which can form a comprehensive evaluation of tourists’ importance and satisfaction with the constituent elements of a destination’s image. The horizontal axis represents importance (I), the vertical axis represents performance (P), and the mean values of the importance and performance indicators are the cutting points of the X and Y axes, respectively [47]. The IPA analysis is mainly divided into four quadrants, I, II, III, and IV [48], which indicate the region of high importance and high satisfaction; the region of low importance and high satisfaction; the region of low importance and low satisfaction; and the region of high importance and low satisfaction, respectively (Fig 2). In this study, the importance indicator *In* is calculated with reference to Eq (2), and the performance indicator Pn is the evaluation score of tourists for the corresponding tourism experience elements. In order to facilitate comparative analysis, this paper takes the lead in drawing quadrant maps of the 12 coastal city destination image perception elements with the help of the IPA model, and finally presents the results centrally. The specific formulas for the calculation of *In* are as follows:

$$In=\left(\frac{Frequency\;of\;occurrence\;of\;element\;n\;in\;the\;text}{Total\;amount\;of\;text}\right)\times 100\%$$ (2)

Where *In* is the frequency of occurrence of the perceived elements of the tourism experience and n is the corresponding element.

### 3.3 Data sources

The data used in this study consisted mainly of travelogues and reviews posted by tourists on the Internet and geospatial data for 12 cities (Table 1).

**Table 1:** Summary of data and sources used in this article.

	Usage data	Data source
User-generated content	Travel reviews and travelogues posted by tourists on travel websites about 12 cities from 2017 to 2021	UGC reviews posted by users on five travel social platforms, including Weibo, Tongcheng, B Station, Mabee’s Nest and Today’s Headlines, from January 1, 2017 to December 31, 2021
Geospatial data	Administrative boundaries of 12 cities	National Earth System Science Data Sharing Platform

With the increasing popularity of the Internet and data processing technology, online big data provides a new channel for studying destination image and tourists’ perceptions, especially Tourist-Generated Content (UGC), such as tourism-related evaluations and travelogues posted by tourists on tourism platforms [49]. Not only can it more fully reflect the perception and feedback of tourists on the destination’s image, but it also puts forward a new research angle and scientific research data for the research of image perception of coastal tourism destinations. In this paper, with the help of a big data information collection system, the UGC comments posted by users on five tourism social platforms, including Weibo, Tongcheng, B Station, Mabee’s Nest, and Today’s Headlines, from January 1, 2017, to December 31, 2021, were collected through searching the keyword “city name + tourism” and an information database was established. Finally, a total of 131,000 texts were collected, and 115,000 remained after elimination, which is the research corpus of this paper. In order to improve the accuracy and credibility of the data, the text content is first pre-processed: ①Delete irrelevant advertisement names, invalid emoticons, etc.; ② Delete media content such as pictures, videos, and advertisements; ③Delete UGC content that has nothing to do with coastal tourism, such as “activity vlogs”; ④Combine synonyms such as “Xinghai” and “Xinghai Bay”; ⑤Create a customized dictionary including the names of coastal city attractions and other words.

## 4. Results and discussion

### 4.1 Coastal tourism network attention

**Fig 3:**
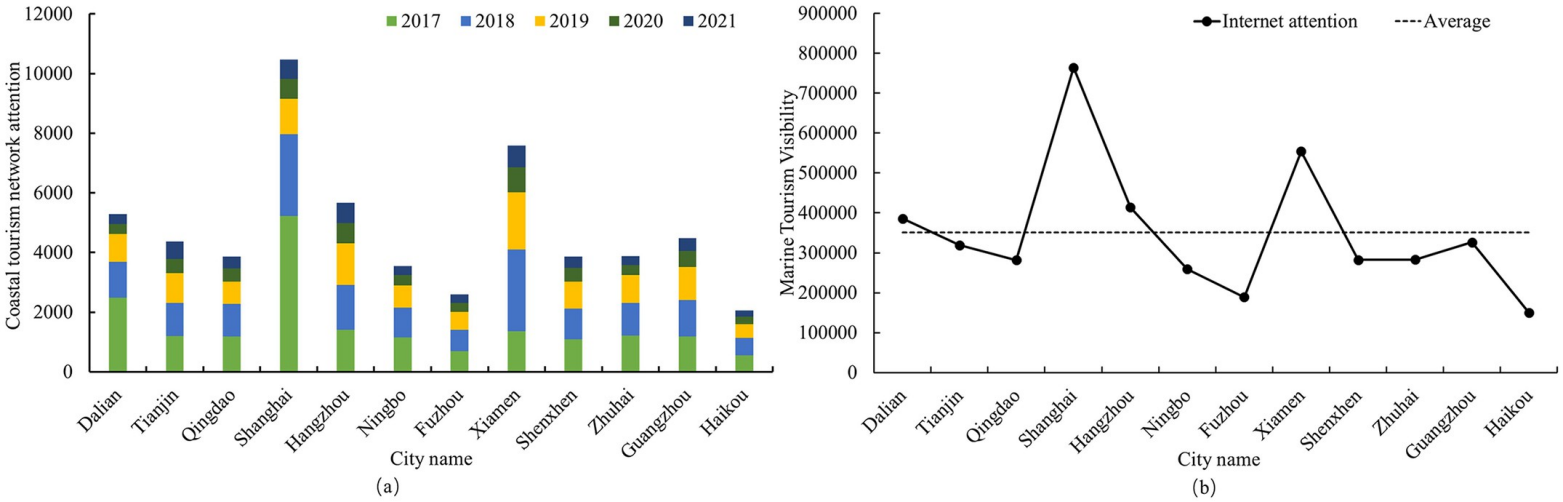
Concern and popularity of China’s coastal tourism network.

Fig 3(a) and 3(b) show the network attention and popularity of coastal tourism in 12 coastal cities. Overall, the network attention to coastal tourism in China shows a downward trend from 2017 to 2021, with 2020 being the turning point. 2017–2019, each coastal city tourism network attention to small changes, and the strength of the network attention between the cities varies, of which Dalian and Shanghai coastal tourism network attention is the highest. In 2020, due to the COVID-19 pandemic, the national tourism industry suffered a setback, and each city’s coastal tourism attention fell sharply. Combined with the results of coastal tourism network awareness in 12 cities, the cities with better performance in coastal tourism network awareness during the study period are Dalian, Shanghai, Xiamen, and Guangzhou, indicating that their coastal tourism image is highly recognized.

### 4.2 Characteristics and difference analysis of the perceived image of coastal tourism destinations

#### 4.2.1 Cognitive image analysis

**Fig 4:**
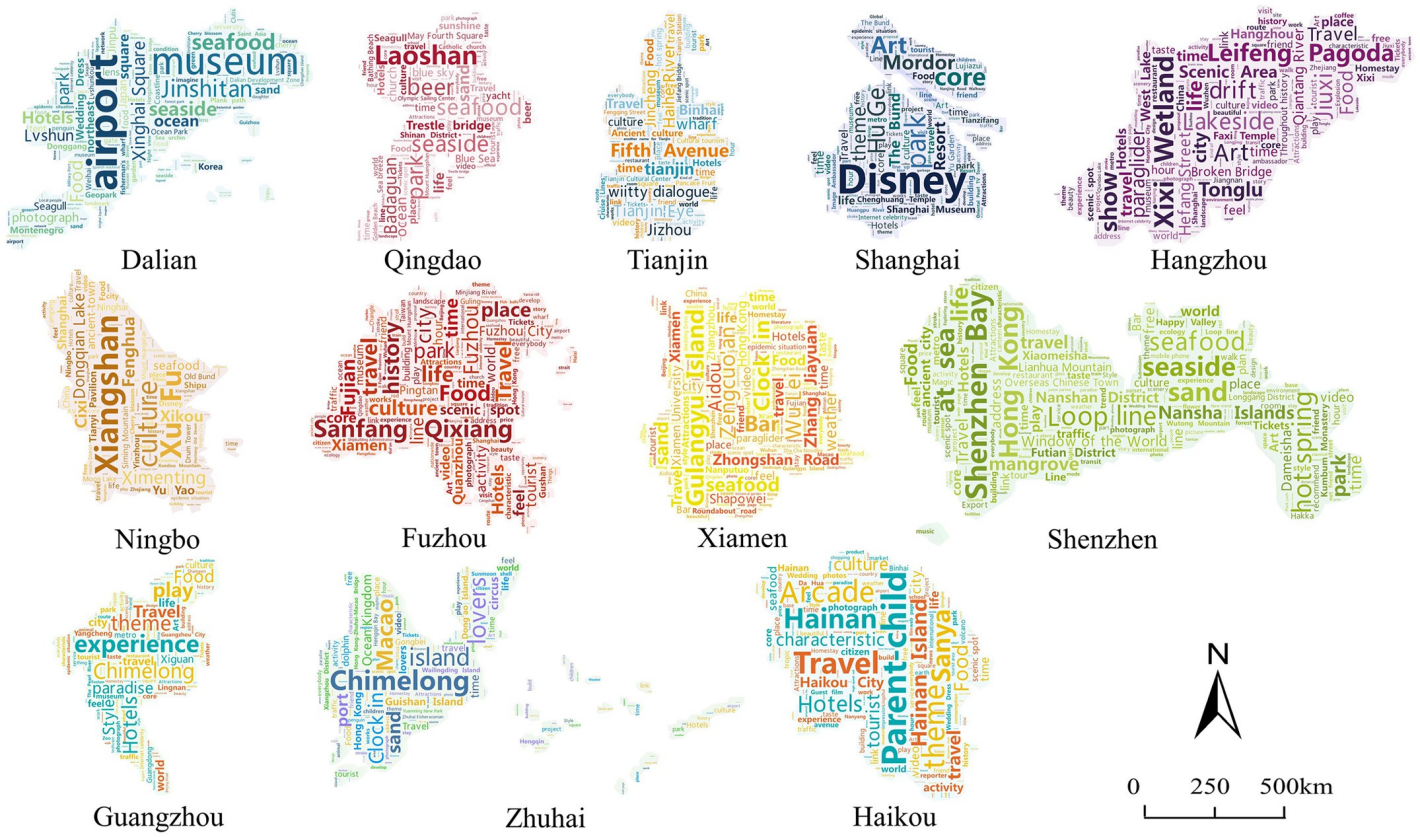
High frequency word cloud map of coastal tourism cognitive image.

Cognitive images are usually analyzed by high-frequency words, and word cloud maps help to make a vivid and intuitive visual presentation. The higher the word frequency, the larger the font and the more conspicuous the color. The actual meaning stands for the tourists’ higher perception and attention to the intended elements and indicates that the characteristics of the tourist resources on which the city is based are more concentrated. Nouns and verbs with a frequency of 200 or more were filtered out, and the high-frequency word maps of the cognitive image of coastal tourism destinations in 12 cities were created based on each city’s word frequency statistics (Fig 4).

From the overall cognitive image perception of coastal tourism, there are certain common and related features in the image perception of coastal tourism destinations by tourists. First, the words “coastal”, “ocean”, “seawater”, and “sea breeze” appear more frequently in 12 cities, including Qingdao (5409), Dalian (4105), and Dalian (4105). Qingdao (5409), Dalian (4105), and Haikou (3015) have the highest frequency of high-frequency words about marine features, indicating that the destination can effectively and fully demonstrate the marine characteristics of the resources. Moreover, tourists pay more attention to the seaside environment of the Bohai Rim and the southeastern coastal areas. Secondly, the high frequency of words such as “hotel”, “food”, “shooting”, “scenic spots”, “accommodation” also indicates that tourists are most concerned about basic tourism elements such as food, accommodation, and transportation while carrying out tourism activities in the destination. Again, due to the geographic proximity of some cities, high-frequency words will be associated, and the cross phenomenon, which is most obvious in the Shanghai-Nanjing-Hangzhou City Circle, such as “Shanghai”, “Disney” and other words also appeared in Hangzhou and Ningbo. This, on the one hand, indicates that these cities have a high degree of cooperation and a strong influence on the coastal tourism of neighboring cities; on the other, it shows that tourists will also pay attention to neighboring destinations.

From the high-frequency word cloud map of the 12 coastal tourism destinations, although they are all based on marine resources, the coastal tourism characteristics of each city are outstanding and distinctly different. According to the word frequency results, the 12 destinations can be divided into four major types. Cities based on marine natural landscapes include Dalian, Qingdao, and Haikou. For example, Dalian’s high-frequency words include “ocean”, “seashore”, “Jinshitan”, etc. Qingdao’s high-frequency words include “green sea”, “sea”, “sea bathing beach”, “seagulls”, etc. Haikou contains “strait”, “holiday beach” and “seafood”. The cultural experience type is represented by Tianjin, Hangzhou, and Fuzhou, such as Tianjin, including mostly “Xiangsheng”, “Jinmen”, “Tianjin Wei”. Hangzhou includes mostly “Song City”, “Ancient Town”, “Hanfu”, etc. and Ningbo includes mostly “Old Bund”, “Old Bund”, “Tianyi Pavilion” and “Nantang Old Street”. Fuzhou includes “Shipbuilding Culture”, “Minhou”, “history” and other words. Tourists show a strong interest in the history and culture of these cities. Leisure and entertainment are represented by Guangzhou, Ningbo, and Shanghai, with high-frequency words such as “theme park”, “art”, “Netflix”, “forest zoo” and so on. Shenzhen, Xiamen, and Zhuhai are mainly tourism and vacation-oriented, with high-frequency words such as “beach”, “hot springs” and “Happy Valley”, representative of a positive vacation and leisure experience for tourists.

(Source of base map: the open source map data service provided by the National Platform for Common GeoSpatial Information Services (https://www.tianditu.gov.cn/))).

#### 4.2.2 Emotional image analysis

**Fig 5:**
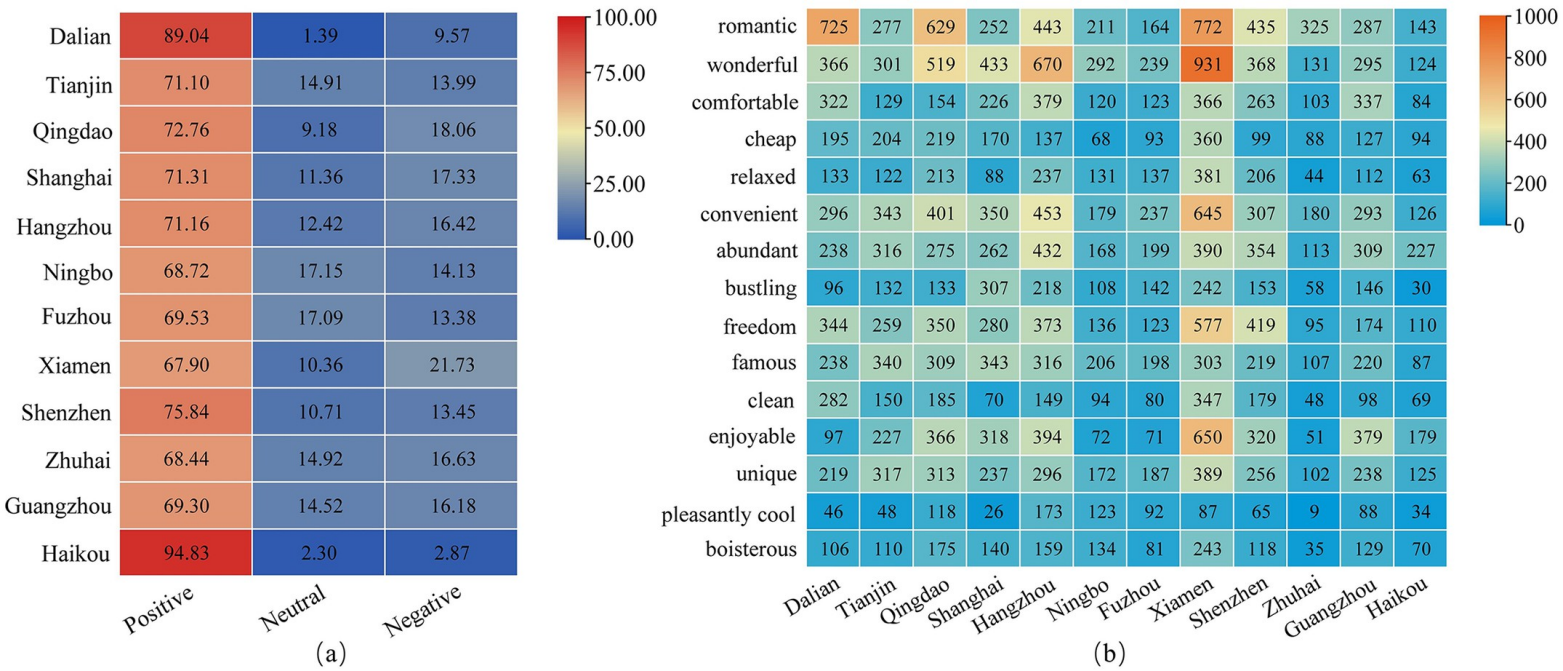
Hot map of emotional image in coastal tourism.

Sentiment analysis examines UGC content using big data algorithms to identify tourists’ sentiment types, mainly positive, neutral and negative. Analyzing the emotional image of each coastal city can provide useful feedback on the tendency of tourists’ emotional attitudes towards coastal tourism destinations. Drawing the heat map of tourists’ emotional preference, based on the statistical results of the proportion of emotional words, (Fig 5(a)) can effectively identify tourists’ emotional attitude bias towards coastal tourism destinations; meanwhile, drawing the heat map of the word frequency of the total high-frequency adjectives in the 12 cities (Fig 5(b)) further explores the difference of tourists’ embodied emotions towards different cities.

From the perspective of general image perception of coastal tourism destinations, tourists’ emotional perception is dominated by positive attitudes, and the average percentage of positive emotions is 74.16%, with positive emotions toward Haikou reaching 94.83% and Dalian reaching 89.04%. Haikou, as a famous coastal tourism destination in China, has a high level of satisfaction among tourists, and Dalian, a famous coastal tourism city in northeast China, also received more positive comments. Based on the positive affective feedback obtained, this paper screened 15 adjectives representing positive emotions that appeared simultaneously in 12 cities to help understand the differences in tourists’ affective perceptions of coastal destinations. First, “wonderful”, “famous”, “unique”, “comfortable”, “joyful”, “cozy” and so on appear frequently, which shows that coastal tourism destinations can provide tourists with a relaxing and lighthearted tourism experience. The word “unique” once more reflects the advantage of coastal cities, represented by their special charm. Secondly, from the perspective of specific cities, tourists’ perceptions of the same emotion words vary in different cities. Among them, in Dalian, Xiamen, Qingdao, and other places “romantic” city atmosphere impressed tourists. Shanghai was described as “convenient”, “prosperous,” “exquisite” and “sophisticated”. Hangzhou’s “simplicity” is also accurately captured. Taken together, travelers’ evaluations of coastal city tourism have a high degree of overlap, but each city has its more prominent and iconic features.

#### 4.2.3 Overall image analysis

**Fig 6:**
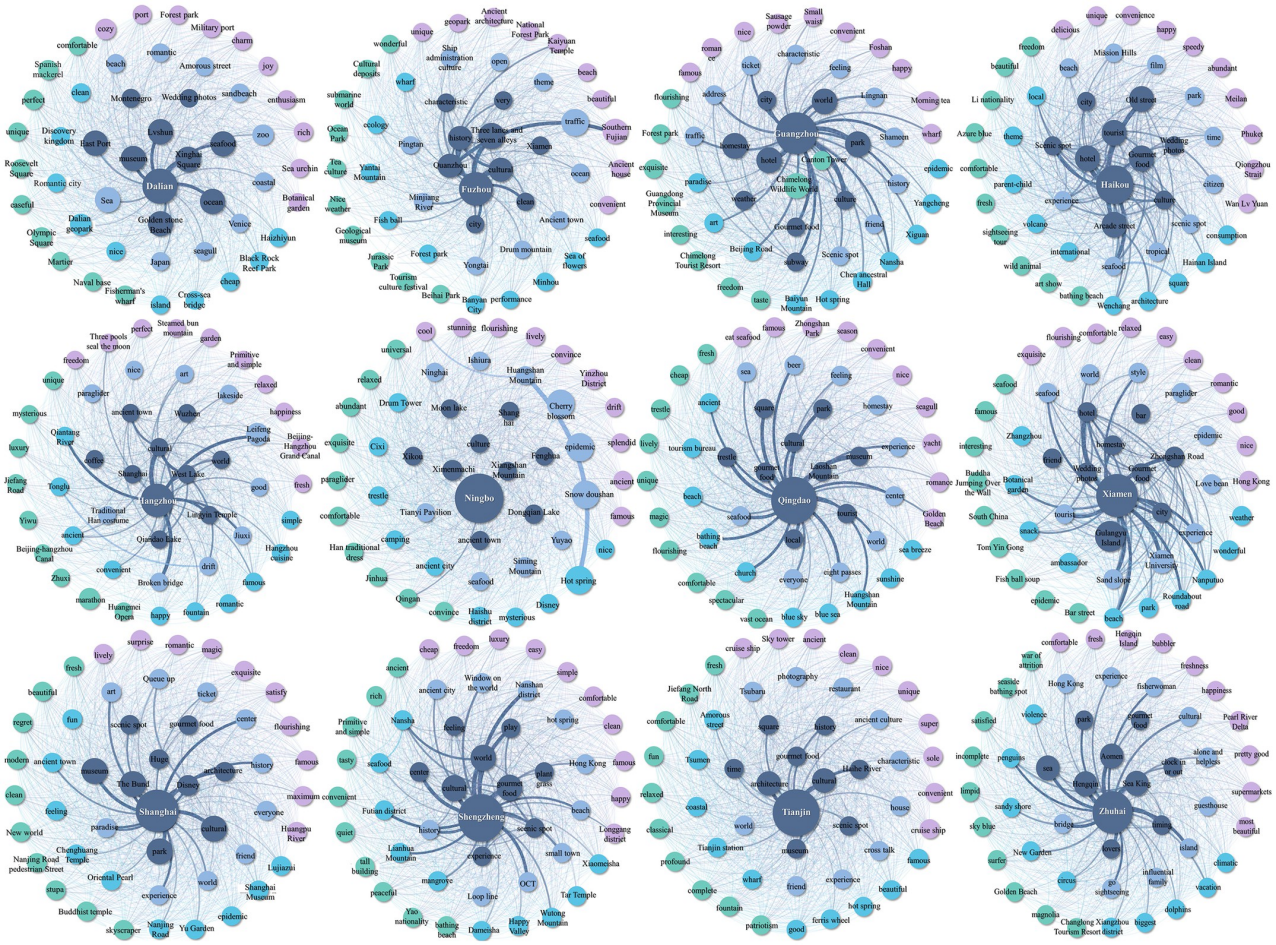
Social semantic network diagram of the overall image of coastal tourism.

To investigate the affinity relationship of tourist attractions in each city, the socio-semantic network of coastal tourism in each city is visualized using Gephi software, with the name of each city as the central word (Fig 6). The socio-semantic network graph expresses the connection between the analyzed elements by the color difference of the nodes, the number of network lines and the degree of thickness. The node color indicates the word frequency of the image elements in the semantic network graph of each city, and the thickness and proximity of the connecting lines of the nodes measure the affinity of each image element. Combined with the semantic map and specific semantics, tourists’ perception of the overall image of coastal tourism destinations can be roughly divided into three circles: “traditional core-characteristic structure-peripheral perception”. The core circle focuses on high-frequency words such as the name of each city and its most representative tourist attractions and facilities that are best known to the general public, reflecting tourists’ deeper cognitive image of coastal tourism resources. The sub-core circle shows the unique tourist attractions of each city; The peripheral circle is mainly composed of words with emotional attitude and little-known tourist attractions, which is an enrichment of the evaluation of the first two circles. The mean value of node relationships of coastal tourism elements in 12 cities is 1105, with small differences in size. It can be seen that there are obvious differences in the comparison of the semantic map of the coastal tourism network of each city. Guangzhou, Qingdao, Shenzhen, Zhuhai, and Haikou present a larger number of semantic network co-occurrences and the relationship between the imagery elements is closer. In contrast, the number of semantic network co-occurrences of Ningbo, Dalian, Tianjin, and Shanghai is smaller, and the relationship between the imagery elements is more distant. Guangzhou, Haikou, and other cities count more famous attractions, and tourists stay longer, so their understanding of the whole city and post-trip evaluations are more adequate. In contrast, the degree of connection between attractions is relatively low, except for some cities that have relatively few famous attractions and, therefore, may receive relatively fewer evaluations from tourists.

### 4.3 Coastal tourism image perception latitude

Perceptual latitude is the main way to measure and evaluate destination images based on high-frequency words and social semantic networks [50]. Through the research and analysis of related literature [51,52], the perceptual elements of the image of coastal tourism are conceptualized and generalized. This process is achieved through manual-aided machine learning: first, more than 1,000 feature patterns suitable for machine learning are manually selected for each category; finally, the remaining patterns are clustered by machine learning. In total, four core categories of tourism resources, tourism services, tourism environment, and tourism society, as well as 20 secondary subcategories (Table 2) were refined to explore further the characteristics of tourists’ perceptions of the image of coastal tourism destinations.

**Table 2:** Perception factor classification of China’s coastal tourism image.

Main category	Subcategory	Corresponding high-frequency words and comments
A Tourist resources	A1 Natural scenery	The metasequoia under the camera is like a painting: dreamy and romantic.
	A2 Scenic spot	The Lingyin Temple in Hangzhou on May Day is really beautiful. There are few people in the West Lake.
	A3 Urban characteristics	#Dalian Tourism # To go to Dalian, female mounted policemen are beautiful scenery.
	A4 Tourism projects	The feeling of jumping from death to life is wonderful.
	A5 Festival activities	Participate in the 2017 Qingdao International Folk Custom Festival at the Olympic Sailing Centre.
	A6 History and culture	A piece of Fuzhou with three lanes and seven alleys, half of China’s modern history.
	A7 Folk customs	The traditional folk culture activity of ‘Fisherman’s Day’ in Lushunkou was held in Beihai Village.
B Tourism services	B1 Food and beverages	After breakfast tea, rice noodles, and desserts, the most important thing is Cantonese food.
	B2 Accommodation services	On the last day of my trip in Guangzhou, I was trapped by a hotel and spent the night at the airport.
	B3 Transportation services	It’s really beautiful to take the Zhuhai sightseeing bus.
	B4 Goods and services	Qingdao duty-free shopping is a new business card of Haikou tourism industry….
	B5 Leisure services	Zhuhai Royal Hot Spring Weekend Tour
C Tourism environment	C1 Seasonal climate	Go to Guangzhou on the 22nd. Now, a super typhoon is approaching.
	C2 Urban sanitation	It’s very clean and convenient. The issue with the third bathroom has been solved.
	C3 Price environment	Children from other places think that consumption in Hangzhou is higher than that in Guangzhou.
	C4 Public administration	#Guangdong found six cases of family aggregation epidemic # Are there any tourists to Guangzhou?
	C5 Marine environment	The seashore of Jinshitan is really vast and suitable for emptying yourself.
D Tourism society	D1 Urban civilization	After seeing Guangzhou promotional film, I know why I don’t want to go.
	D2 Social atmosphere	The people of Fujian are kind and lovely, except for some.
	D3 Tourism practitioners	The tour guide on the sightseeing bus recommends a one-day tour in Lushun, and the route is too deep.

### 4.4 IPA analysis of image perception of coastal tourism

The UGC data published by tourists are the subjective expression of tourists’ perceptual experiences of coastal tourism destinations, and according to the generic category of the elements of image perception of coastal tourism destinations, we can assign further points to tourists’ subjective sentences on each perceptual element [53]. First, the subjective sentences for the previous classification and subclasses are organized. Second, the five-level Richter scale (1 = very poor; 5 = very good) allows tourists to assign the value of a-e points. Finally, the satisfaction scores were summarized and calculated. The process was completed by four coders who independently assigned scores to the texts and recoded the texts when the comparison results were different until they were consistent.

The coding process is as follows.

UGC Sample: “Dalian is a good place, not too cold in winter and not too hot in summer, and the Lotus Hill Observation Deck is so nice! Taking the Haida ropeway up the mountain saves the most energy, and the view from the ropeway is great”. The corresponding code is: “C1b; A2a; A4a;”, where the code “A1a” indicates that the image perception element in the sample is “tourist attractions” and the emotional expression of satisfaction is “Very good”.

**Fig 7:**
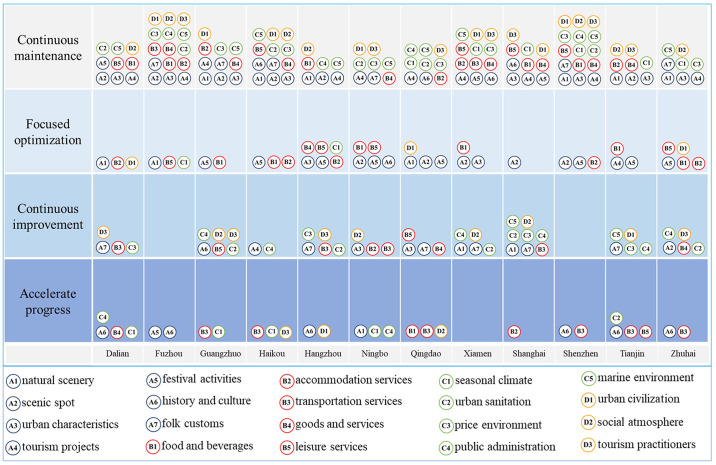
Analysis of IPA values of perceived elements of coastal tourism destination terrain images.

The results of the IPA analysis are shown in Fig 7. The elements in Quadrant I (Continuing Maintenance Zone) indicate that the elements of the city’s perceived image of coastal tourism have a high degree of importance and strong satisfaction; the elements falling in Quadrant II (Focused Optimization Zone) indicate a lower degree of attention but a high degree of satisfaction, Quadrant III (Continuous Improvement Zone) indicates that these elements have a low degree of attention and a low degree of satisfaction. Quadrant IV (Accelerated Advancement Zone) indicates that the elements exhibit high levels of concern but low levels of visitor satisfaction.

1. Tourism resources. Tourism resources support tourists in completing tourism activities and are at the core of the construction of the coastal tourism network image’s perceived elements. During the study period, the overall perceived satisfaction with tourism resources was relatively high, with more than half of resources distributed in the Quadrant I and secondary elements unevenly distributed among cities with some emphasis. The five perceptual elements of natural scenery, tourist attractions, urban characteristics, tourism projects, and folk customs were distributed in more than half of the cities in Quadrant I, with festivals and events occupying eight cities in Quadrant II. This shows that tourism practitioners in these cities can effectively promote the incorporation of core tourism resources, and a better tourism experience can meet tourists’ expectations, as well as contribute to the shaping of the destination’s tourism image. Further, this also allows for the perception of better performance to fully grasp the advantageous resources, to further explore the city’s characteristics to attract, and to play a leading role in the advantageous resources. History, culture, and folklore were distributed in five cities in Quadrants III and IV. Perhaps tourists do not travel to the destination with the original goal of experiencing the relevant elements, but these elements unsatisfactory tourism experiences. The analysis found that in some of the cities the use of historical and cultural projects failed to deal with the relationship between the national cultural background and the need to properly cultivate the unique charismatic cultural connotation to avoid weakness. The analysis shows that some cities have failed to address the relationship between the national cultural background when using historical and cultural projects. In terms of cities, Haikou is the most satisfied with the image of coastal tourism network, with the most perceived elements in Quadrant I, followed by Dalian, Fuzhou, Guangzhou, Shanghai, Shenzhen and Zhuhai, which shows that most cities can effectively utilize these tourism resources in the process of tourism development and that tourist perception and destination image are better formed. Ningbo, Qingdao, Xiamen, and Zhuhai need to better explore their urban characteristics to optimize the elements.
2. Tourist services. Tourist services, as a guarantor in the process of tourist activities, are an important competition element for service quality in the destination’s tourist image. Overall, they are mainly distributed in Quadrants I, II, and the number of cities with hospitality and merchandise services in Quadrants I is 10 and 9, respectively. This indicates that the cities have done a better job in these areas, resulting in higher tourist satisfaction. Six cities with accommodation services are in Quadrant II, indicating that accommodation is not the focus of tourists’ attention when perceiving the destination, but their level of service is recognized by tourists. Visitors’ perception of transportation services is negative, with four cities in Quadrant III and six in Quadrant IV. Congestion, urban traffic accidents and control chaos are common, suggesting that management needs to pay attention to these issues and resolve them promptly. From a city perspective, tourists perceive Fuzhou, Xiamen, and Shenzhen as the most positive and top-rated cities in terms of tourism services, followed by Hangzhou and Zhuhai. The management of the remaining cities needs to focus on optimizing the places with low satisfaction. Cities need to improve their tourism spaces and services to promote the development of tourism activities.
3. Tourism environment. The basic environment for tourism activities includes not only the ever-changing natural environment but also the healthy and intact public environment, which plays a crucial role in the survival and development of a particular destination’s tourism. Overall, the results of the perception of the tourist environment’s elements are more evenly distributed among the four main elements of perception, which also indicates that the performance of the natural and social environment of coastal towns is very different. In terms of natural environment, as the core element of coastal tourism, marine environment shows the best positive attitude among tourists, with ten cities in Quadrant I, indicating that the unique marine environment of these cities can provide tourists with an excellent tourism experience. China’s coastal regions are rich in climate types. Although extreme weather and temperature differences may sometimes affect tourism activities, eight cities are located in Quadrants I and II, while Dalian, Hangzhou, Haikou, and Guangzhou are situated in Quadrant IV, which requires timely improvement of cities’ prevention and control measures. More than half of the cities in the social environment are in Quadrant I for health and price environment, with better performance in terms of visitor perception. Guangzhou, Hangzhou, Xiamen, Tianjin, and Zhuhai need to be emphasized and improved by relevant managers. Due to the Xin Guan epidemic, most of the cities were in Quadrants III and IV in terms of public administration during the study period. Cities need to optimize and improve prevention and control measures to strengthen the protection of tourism activities.
4. Tourism society. Tourism society, which we could say acts as a city’s business card, can show the comprehensive strength of urban culture, essential to the optimization of the destination image. Overall, most of the elements are distributed in Quadrant I, indicating that it is possible to create strong synergies to improve a city’s civilization level,, urban publicity, and a good social atmosphere to enhance the sense of well-being of tourists traveling. In terms of the city’s public relations and branding compared to other cities, Hangzhou is more affected by inappropriate publicity and public incidents and needs to take advantage of the media to improve its marketing strategies. Guangzhou, Ningbo, Xiamen, and Shanghai need to strengthen urban cohesion and the citizens’ sense of responsibility, providing tourists with a friendly atmosphere that makes them feel at home.

In general, the perceptual elements of Chinese coastal tourism have a high degree of expressiveness, most of which are in the medium or higher satisfaction range. They are mainly concentrated in Quadrants I and II, with a few in Quadrant III, indicating that China’s coastal tourism destinations have a better level of image building overall, and that they have done a lot of work in terms of attracting tourists’ attention and better satisfying their needs. However, at the same time, attention must be paid to the four perceived elements: improving satisfaction with the tourism environment, improving tourism infrastructure and supporting facilities, improving environmental standards, completing the tourism function, developing the quality of tourism services, and promoting the rapid development of the coastal tourism industry.

## 5. Implications

### 5.1 Theoretical implications

Four main points in this study will contribute theoretically to future research on tourist destination image perception. First, this study explores the image of coastal tourism destinations from the perspective of tourists, taking 12 coastal cities as examples, which is an enrichment of the research results of city image theory and psychology’s “cognitive-emotional” theory. Research on the attractiveness and competitiveness of coastal tourism cities is mainly based on the unique endowment of coastal tourism resources, while most previous studies on destination image regard coastal cities as general destinations [54,55]. On the other hand, from tourism perception to marketing design, the core subject of coastal tourism activities is the tourists, which essentially requires a combination of tourists’ perceptions and local resource characteristics [56]. Therefore, this study complements the tourism image of coastal cities from the perspective of tourists and puts forward reasonable marketing suggestions, providing a theoretical basis for the sustainable development of coastal city tourism.

Second, although existing studies try to explore the image of coastal tourism, most of the case studies on the perception of coastal tourism destinations are based on specific scenic spots or cities [57,58], so the conclusions drawn are somewhat limited. This study expands the research selection to 12 typical coastal tourism cities with different styles, which on the one hand can discover the commonality of coastal cities’ tourism image, and on the other hand explores the differentiated perceptions that the 12 cities bring to tourists. This is an important reference value for coastal tourism cities to enhance their competitiveness from differentiation while relying on homogenized tourism resources.

In addition, this study categorized the factors affecting the perceived image of coastal tourism, focusing on the elements of destination perception. Although many scholars have studied the factors affecting tourists’ perceptions [59], and the research on the factors affecting the perception of individual coastal tourism destinations has also been developed, there is a lack of a universally applicable reference scale for these influencing factors. Therefore, this study summarizes the potential variables as the core factors affecting tourists’ perception of coastal tourism destinations as a reference for future research in this field.

Finally, this study attempted to reduce human bias in the research process through targeted mining of web text data using machine learning and independent coding. Most research on tourism destination image perceptions combines qualitative and quantitative methods, meaning that the researcher’s preferences may influence the results. For example, a researcher conducted a comparative study of tourists’ and residents’ destination image perceptions through accompanying photo interviews and questionnaires, but found that they essentially experienced the same perceptions [60]. Therefore, the current study’s approach to scientifically reducing human error using machine learning can be regarded as a reference for subsequent studies.

Taken together, as tourism is a psychosocial phenomenon [61], the study of tourist place perceptions aims to explore this psychosocial phenomenon in depth. Subsequent research on tourism behavior should not only be theoretical and limited to the perception of coastal cities’ tourism images but it also requires the academic community to conduct deeper practices on its characteristics and mechanisms through empirical research.

### 5.2 Practical implications

In practice, tourism destination management and sustainable development must study people’s tourism experiences in destination cities in the context of the experience economy [62]. Therefore, this paper can help China’s coastal cities to clearly understand tourists’ cognitive overview of each coastal tourism city’s image so as to determine their own tourism positioning. At the same time, through comparative analysis, each coastal city can determine its own tourism competitive advantages compared to other cities, therefore expanding its advantages and compensating for its disadvantages.

From the perspective of enhancing tourist attachment, tourists develop initial impressions before, in-depth impressions during, and actual impressions after a said tourism activity [63]. UGC data’s open and free nature allows travel portals to create a “parallel space” for interaction between potential and actual tourists. This space provides a clearer picture of how a destination is portrayed during the visitor experience while enhancing word-of-mouth related to the destination. For coastal tourism marketing agencies, the tourist experience in the process of going deeper into a destination is the main tourism product and service. Tourism image marketing and tourist perceptions are dynamic processes that interact with each other [64]. The more a region’s destination image is unique, the likelier it is to thrive in the highly competitive tourism industry. Therefore, people providing tourism services should focus on the coastal tourism products themselves, optimize the infrastructure, improve the natural environment, and highlight special touring and experiential products, thereby creating core highlights and major competitiveness.

In terms of coastal tourism target cities, maintaining a good tourism image is required to ensure a steady flow of tourists and generate related revenue. In addition to tourists’ strong emotional tendencies and willingness to travel to a particular destination, a destination’s image becomes a key factor influencing trip choices. During tourism activities, tourists gain insight into local customs, transport prices, and social atmospheres. Through travelogues and comments posted by tourists, cities can effectively understand their allocation of tourism resources, further improve tourism facilities, and enhance comprehensive tourism services. Cities can also improve their visibility through large international events, improve their tourist destination image, and show their comprehensive strength.

The shaping and maintenance of a destination image is a long process requiring perseverance and investment. Coastal areas have unique geographical advantages and tourism resources setting them apart from inland areas; therefore, they should publicize their individuality and vitality. Based on careful consideration of the tourism experience of the target market, each region must clarify its own urban image positioning, examine each city’s characteristic culture, highlight the coastal tourism charm, and emphasize the spread of the diversity of coastal tourism resources, improving tourists’ satisfaction and forming a lasting attraction.

## 6 Conclusions and limitations

### 6.1 Conclusions

The study of coastal tourism image perception stems from the basic need for a positive image design and efficient marketing strategy; at the same time, it plays a guiding role in the planning and development of coastal tourism resources. In this paper, we used a text collector to obtain the UGC text of tourists’ tourism experience in Chinese coastal cities in five years. By using software and methods such as Gephi, Tbtools and jieba corpus analysis, IPA modeling, etc., we conducted an in-depth study on their cognitive-emotional image, overall image, and factors influencing the perceived image. We found that the construction of the network image of each coastal tourism city in China was preliminarily formed. It was found that the network image construction of each coastal tourism city in China has taken shape, that tourists are more optimistic about the image of coastal tourism destinations, and that the perceived image factors of each coastal tourism city differ in terms of satisfaction and other characteristics. The conclusions are as follows:

1. Coastal city tourism network attention in the study period showed a downward trend. Tourism activities are more vulnerable to external factors. Destinations need to adapt to the times and actively adjust the relevant policies. Among the twelve coastal cities, Shanghai and Xiamen have the highest tourism visibility, while Fuzhou and Haikou should actively conduct tourism promotion activities to enhance their tourism appeal.
2. The characteristic image of the “ocean” of the 12 coastal cities can be better perceived by tourists, among which the Bohai Rim and the southeast coast receive higher attention. The basic elements of tourism, such as food, accommodation and transportation, are the key elements that tourists prioritize when carrying out tourism activities, and it is necessary for destinations to strengthen and improve the construction of tourism infrastructure. Although all marine resources are attractive, each city’s appeal differs significantly for tourists. The experience of coastal tourism in Shanghai, Ningbo, and Hangzhou shows a high degree of consistency, and strengthening regional cooperation is also an effective channel to develop coastal tourism. Overall, tourists have a positive attitude towards the tourism image of China’s coastal cities, and there is a high degree of adjectival overlap in positive affective evaluations. However, the degree of warmth of the overlapping adjectives varies across cities, and tourists’ affective experience varies from city to city.
3. The socio-semantic network diagrams of the tourism image of each coastal city are presented in three circles, including “traditional core-characteristic construction-peripheral cognition”, but the specific contents are different. This not only shows that the tourism characteristics of each coastal city in China are distinct but also indicates that deficiencies still exist in the improvement of service facilities and the acceleration of culture and tourism integration. There is still room for progress in the brand image building of coastal tourism cities, and the goal of maximizing the display of coastal tourism characteristics according to the local conditions is still to be accomplished.
4. The perceived elements of coastal tourism image are categorized into four main categories and 20 subcategories, namely tourism resources, tourism services, tourism society, and tourism environment, with tourists’ satisfaction varying with each perceived element across cities. In general, satisfaction with the perceived image of coastal tourism destinations is better, with tourism resources and tourism environment more distributed in the entertainment area, indicating that coastal tourism resources are abundant in each city, and most tourism resources and tourism environment bring tourists a better experience and more satisfaction. Tourism services and the social elements of tourism are not uniform in the distribution of the four quadrants. Regardless of whether the focus is on the tourist experience for tourists, the management and marketing of tourist destinations should focus on improving the construction of tourist facilities, creating a good social atmosphere, and actively and reasonably publicizing the image of the city to improve the charm of the coastal tourist attraction.

### 6.2 Limitations and future research

This study mainly discusses differences in image perceptions of China’s coastal tourism cities; this means it presents deficiencies and limitations that should be improved in future research. First, this study employed many case studies, but the data collected from each case differ; therefore, results of a text analysis of individual cities with large amounts of data would be richer and more diverse. Second, this paper only uses text data; however, with the expansion of visual sharing channels publishing short videos and pictures, the sample space could be expanded by adding images, instant bullet screens, and other content. Finally, the particularity of coastal tourism resources affects its impact factors, so the impact factors of coastal tourism cities’ image perception require further exploration.

## Supporting information

S1 Fig China coastal tourism cities network attention. (JPG)

S2 Fig Network visibility of China’s coastal tourism cities. (JPG)

S3 Fig Percentage of tourists’ emotional attitudes toward China’s coastal tourism cities. (JPG)

S4 Fig Word frequency statistics of positive affective adjectives in Chinese coastal tourism cities. (JPG)

S1 Dataset China coastal tourism cities network attention. (XLSX)

S2 Dataset Network visibility of China’s coastal tourism cities. (XLSX)

S3 Dataset Percentage of tourists’ emotional attitudes toward China’s coastal tourism cities. (XLSX)

S4 Dataset Word frequency statistics of positive affective adjectives in Chinese coastal tourism cities. (XLSX)

S5 Dataset Statistics of high frequency words in China’s coastal tourism cities. (XLSX)

S6 Dataset Statistical results of tourists’ IPA evaluation of coastal tourism in Chinese cities. (XLSX)
