# Supplementary figures and images for: Perceived differences in coastal tourism image under tourist experience-IPA analysis based on UGC data of 12 coastal cities
Source: PLoS One. 2024 Aug 22;19(8):e0299431. doi: 10.1371/journal.pone.0299431 (PMC11341062; doi:10.1371/journal.pone.0299431)

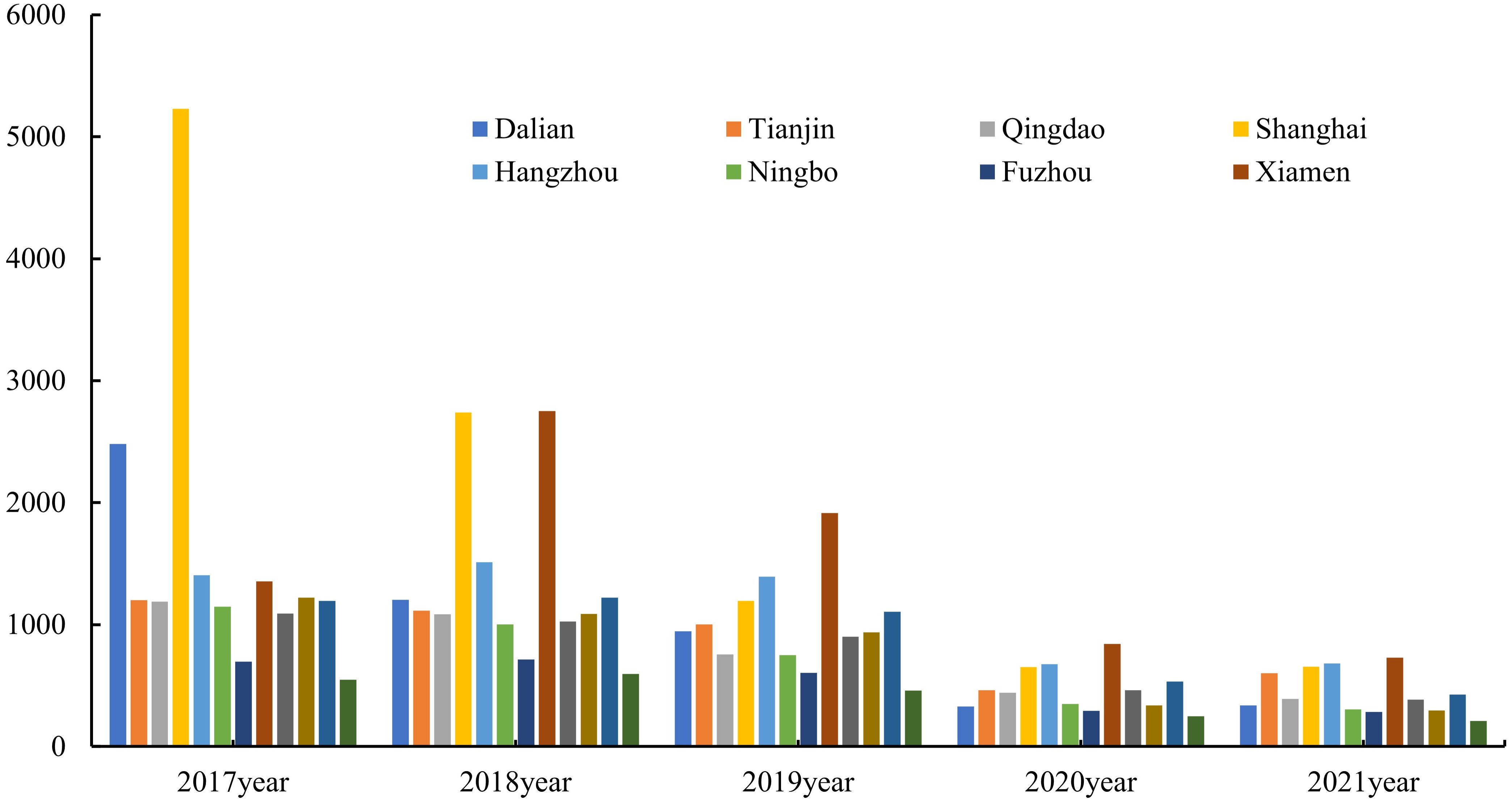

Supplement: S1 Fig — (JPG) [file pone.0299431.s001.jpg]

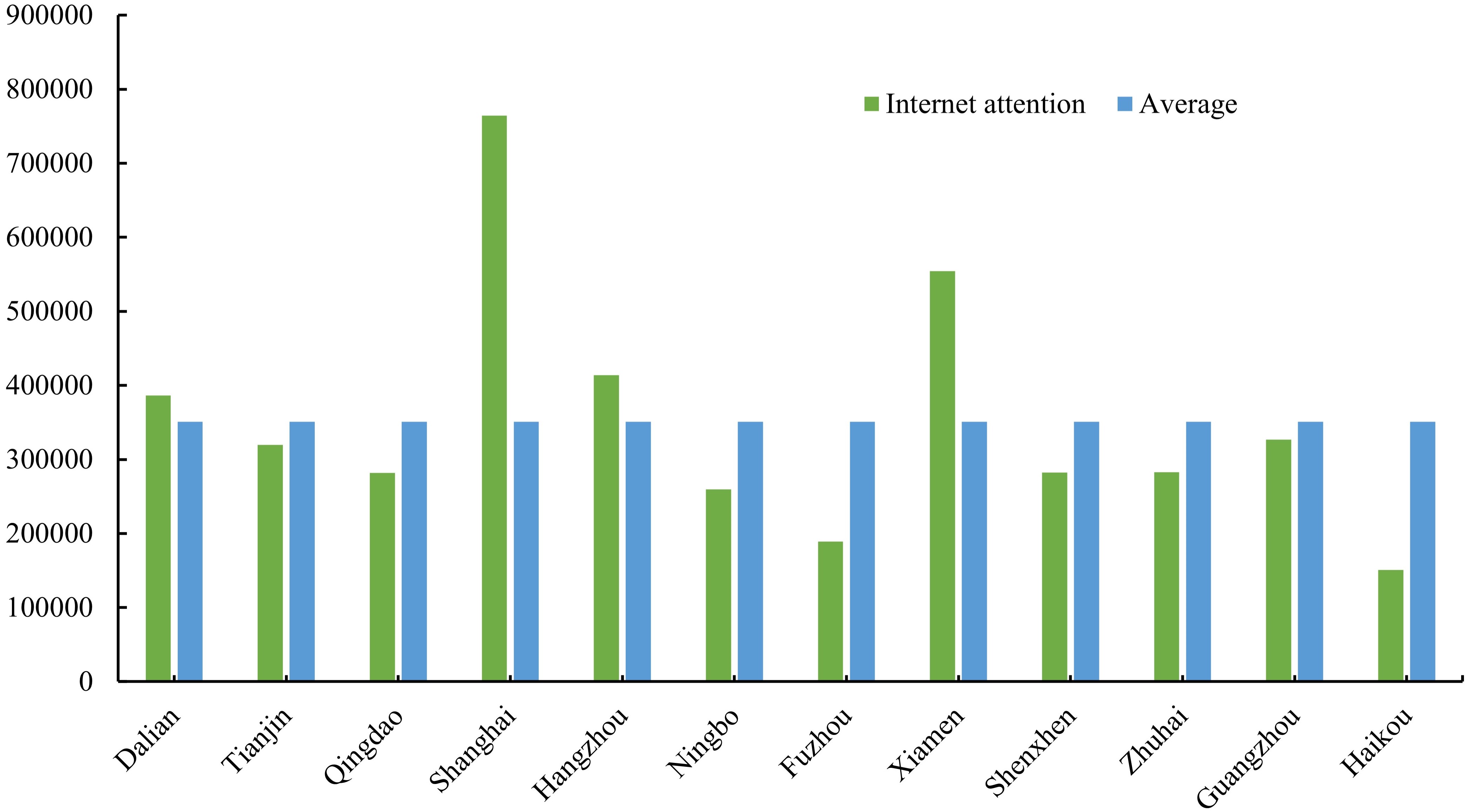

Supplement: S2 Fig — (JPG) [file pone.0299431.s002.jpg]

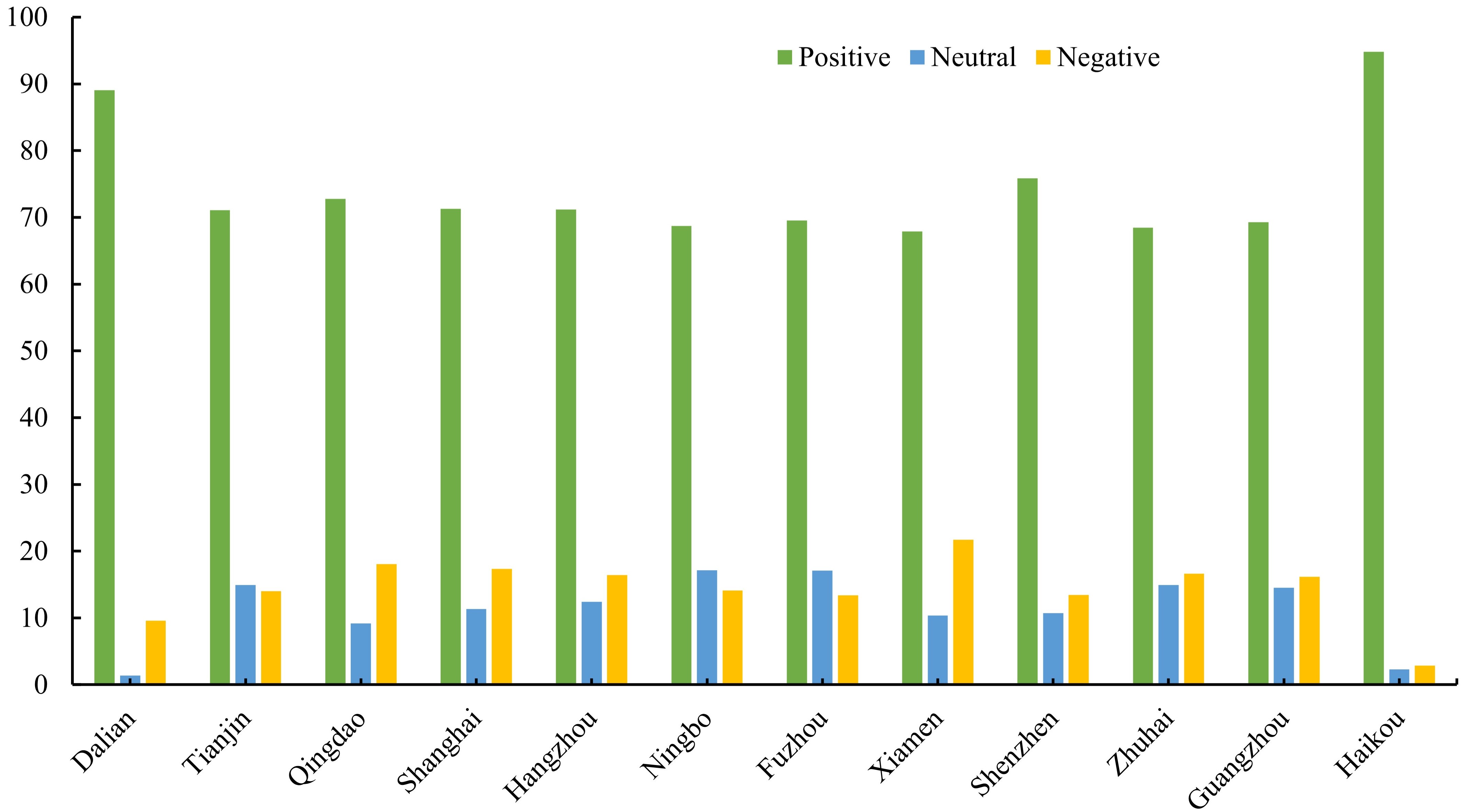

Supplement: S3 Fig — (JPG) [file pone.0299431.s003.jpg]

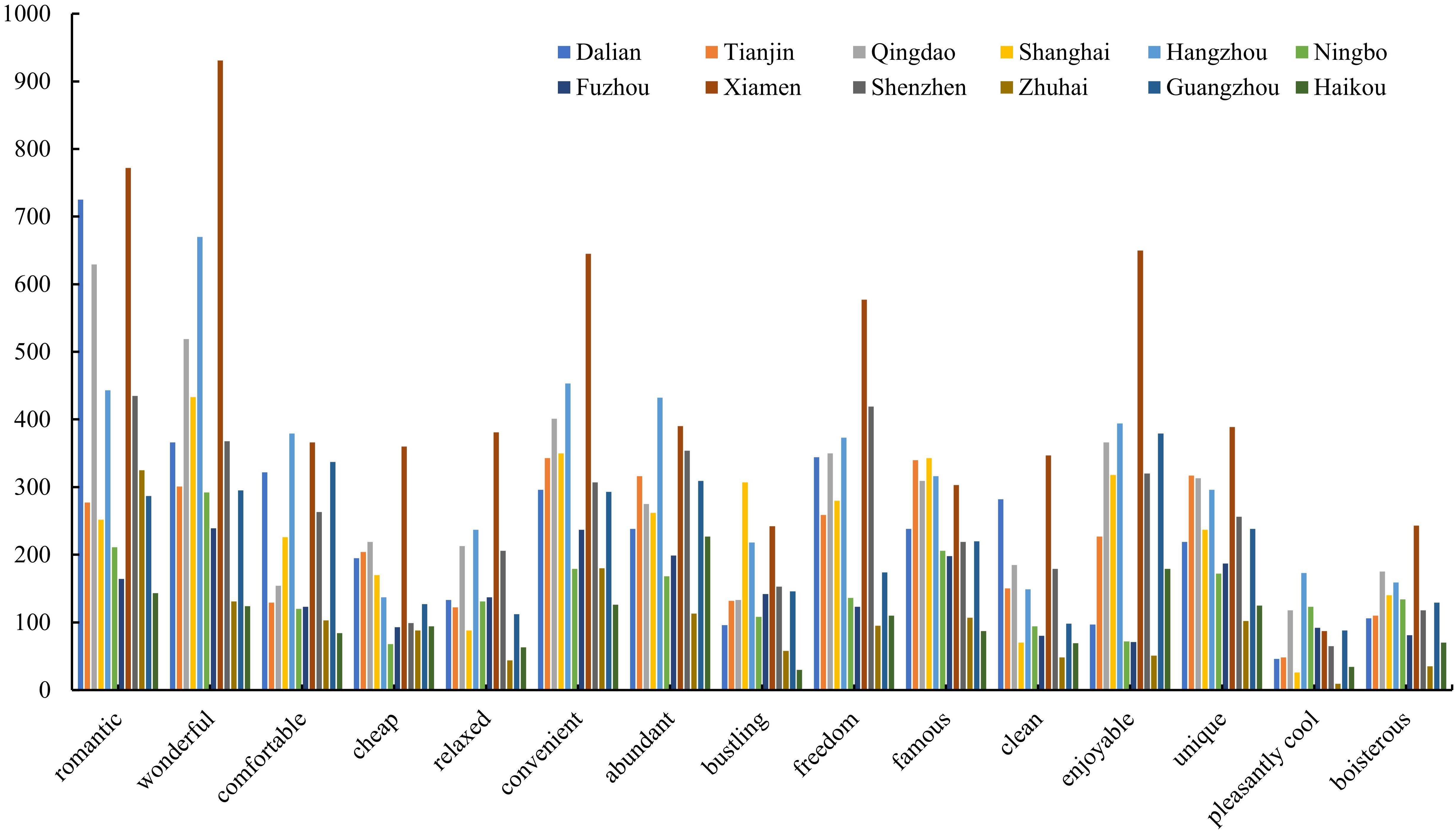

Supplement: S4 Fig — (JPG) [file pone.0299431.s004.jpg]
